# Hematological Toxicities with PARP Inhibitors in Prostate Cancer: A Systematic Review and Meta-Analysis of Phase II/III Randomized Controlled Trials

**DOI:** 10.3390/cancers15194904

**Published:** 2023-10-09

**Authors:** Gartrell C. Bowling, Piragash Swargaloganathan, Carly Heintz, Ravi A. Madan, Binil Eldhose, Albert Dobi, Gregory T. Chesnut

**Affiliations:** 1 School of Medicine, Uniformed Services University of the Health Sciences, Bethesda, MD 20817, USA; 2 Center for Prostate Disease Research, Murtha Cancer Center Research Program, Department of Surgery, Uniformed Services University of the Health Sciences, Bethesda, MD 20817, USA; 3 Genitourinary Malignancy Branch, Center for Cancer Research, National Cancer Institute, National Institutes of Health, Bethesda, MD 20892, USA; 4 Henry M Jackson Foundation for the Advancement of Military Medicine, Inc., Bethesda, MD 20817, USA; 5 Urology Service, Walter Reed National Medical Center, Bethesda, MD 20814, USA

**Keywords:** Poly ADP-ribose polymerase (PARP), PARP inhibitors, olaparib, veliparib, rucaparib, niraparib, talazoparib, prostate cancer, CRPC

## Abstract

**Simple Summary:**

Poly ADP-ribose polymerase inhibitors (PARPis) can be used in men with advanced prostate cancer. Unfortunately, this class of therapeutics has demonstrated the risk of developing hematological adverse events. We conducted a systematic review and meta-analysis of several PARPi clinical trials to further evaluate the incidence of anemia, thrombocytopenia, and neutropenia adverse events in prostate cancer patients. We found that this treatment is significantly associated with hematologic suppression. Further stratification suggests differences may exist between PARPis within this class of therapy.

**Abstract:**

Background: Poly ADP-ribose polymerase inhibitors (PARPis) are an important class of therapeutics for metastatic castration-resistant prostate cancer (mCRPC). Unlike hormone-based treatments for mCRPC, PARPis are not without drug-related hematological adverse events. Objective: To review the evidence on hematological toxicities, including anemia, thrombocytopenia, and neutropenia from PARPis in prostate cancer. Study Methodology: A systematic review and meta-analysis using the PRISMA guidelines was performed for phase II and III randomized controlled trials (RCTs) of PARPis in prostate cancer. PubMed, Embase, and Ovid All EBM reviews—Cochrane were queried from inception to 9 June 2023. The Mantel–Haenszel method was used to report risk ratios (RR) and 95% confidence intervals (CI) for all-grade and high-grade anemia, thrombocytopenia, and neutropenia toxicities. Results: The systematic review retrieved eight phase II and III RCTs; specifically, eight were included in the anemia, five in the all-grade thrombocytopenia and neutropenia, and four in the high-grade thrombocytopenia and neutropenia outcomes. Compared to a placebo and/or other non-PARPi treatments, PARPi use was associated with an increased risk of all-grade anemia (RR, 3.37; 95% CI, 2.37–4.79; *p* < 0.00001), thrombocytopenia (RR, 4.54; 95% CI, 1.97–10.44; *p* = 0.0004), and neutropenia (RR, 3.11; 95% CI, 1.60–6.03; *p* = 0.0008). High-grade anemia (RR, 6.94; 95% CI, 4.06–11.86; *p* < 0.00001) and thrombocytopenia (RR, 5.52; 95% CI, 2.80–10.88; *p* < 0.00001) were also associated with an increased risk, while high-grade neutropenia (RR, 3.63; 95% CI, 0.77–17.23; *p* = 0.10) showed no significant association. Subgroup stratification analyses showed differences in various all-grade and high-grade toxicities. Conclusion: PARPis were associated with an increased risk of hematological AEs. Future studies with more pooled RCTs will enhance this understanding and continue to inform patient–physician shared decision-making. Future studies may also have a role in improving the current management strategies for these AEs.

## 1. Introduction

Prostate cancer (PCa) management has rapidly changed over the past few decades due to significant advancements in screening, treatment, and understanding of genomics underlying the disease. As the most common non-cutaneous malignancy diagnosed in men, this cancer remains a leading cause of male-specific cancer deaths globally [1]. Fortunately, in developed countries, most cases of PCa are detected in the early stages and are amenable to active surveillance, curative intention surgery, or radiation therapy [2]. Second-generation androgen receptor axis inhibitors (ARAI) are the mainstay treatment for most advanced forms of PCa. However, these treatments are not curative, and most patients are likely to develop metastatic castration-resistant prostate cancer (mCRPC), a resistant subset of tumor that no longer responds to treatments targeting the androgen receptor axis [3]. This becomes a challenge for medical professionals when it comes to improving patient survivability and quality of life [4,5,6]. Poly ADP-ribose polymerase (PARP) inhibitors, with multiple agents approved across therapy lines and in combination regimens, are now a well-established treatment option for men with mCRPC [7,8,9].

PARPs, a family of 17 multi-functional enzymes, play essential roles in DNA damage repair via homologous recombination repair (HRR) and base excision repair. While not necessary for HRR, PARP-1 appears to recognize and facilitate single stranded break (SSB) repairs [10]. As such, there is also a plausible role in double stranded breaks (DSBs) [11]. However, inhibition of PARP-1 in patients with concomitant HRR alterations, specifically BRCA 1/2, promotes lethal DSBs and subsequent cell death [12,13,14,15]. In 2015, Robinson et al. demonstrated the high prevalence of HRR aberrations in mCRPC [16]. Soon after, Mateo et al. showed PARP inhibition produced an overall clinical improvement in mCRPC patients with HRR mutations [17]. Since then, PARP inhibitors (PARPis) have received major attention for optimizing mCRPC management [14,18,19].

Currently, there are four Food and Drug Administration (FDA)-approved PARPis for patients with HRR-positive mCRPC: Olaparib, Rucaparib, Niraparib, and Talazoparib [20,21,22,23,24,25,26]. Veliparib is currently under investigation [20,27]. Like all cancer drugs, PARPis are not without adverse events (AEs). Hematological toxicities, including anemia, thrombocytopenia, and neutropenia, are among the most common AEs reported in PARPi treatments. Previous systematic reviews and meta-analyses of PARPis in pooled cancers have reported high incidences of hematological toxicities [28,29,30,31,32]. A recent review looking at the FDA Adverse Event Reporting System (FAERS) database encompassing primarily ovarian cancers reported an association of PARPi treatments with early onset of hematological toxicities [32]. Unfortunately, in some patients, these AEs become too severe, requiring treatment dose modifications or a temporary discontinuation. Clinicians must comprehend these AEs and engage in shared decision-making with patients, considering their clinical characteristics and treatment objectives. 

Given this well documented risk, we conducted an up-to-date meta-analysis of eight phase II and III PCa PARPi randomized controlled trials (RCTs) to determine the incidence and risk of hematological AEs with PARPis. To the best of our knowledge, there does not exist a dedicated analysis evaluating these hematologic AEs in mCRPC. Furthermore, in the setting of several published PARPi trials in the year 2023 along with recent FDA approval of Olaparib, Niraparib, and Talazoparib, this up-to-date study provides contemporary analyses of these hematologic AEs [21,25,26,33,34,35,36]. 

## 2. Materials and Methods

### 2.1. Search Strategy

This systematic review and meta-analysis was undertaken following the RCT guidelines in the Preferred Reporting Items for Systematic Reviews and Meta-Analyses (PRISMA) [37]. See Supplementary Table S1 for the PRISMA checklist. The study was registered with the International Prospective Register of Systematic Reviews (PROSPERO registration: CRD42023426201) in May 2023. The following databases were searched from inception to 9 June 2023 to identify RCTs with PARPi in PCa: PubMed, Embase, and Ovid All EBM reviews—Cochrane. The search was limited to the English language. The following search terms were used: “PARP” AND “olaparib OR veliparib OR rucaparib OR niraparib OR talazoparib OR pamiparib OR fuzuloparib” AND “prostate cancer” AND “randomized controlled trial OR clinical trial”. See Supplementary Table S2 for the detailed search strategies. Additional manual searches were conducted by reviewing pertinent reference lists and using common internet search engines with the above terms.

### 2.2. Selection Criteria

The inclusion criteria were based on the PICO framework. Population (P): prostate cancer patients. Intervention (I): treatments containing a PARPi. Comparison I: placebo or treatments without a PARPi. Outcomes (O): hematological toxicities, including anemia, thrombocytopenia, and/or neutropenia. The studies included were published phase II and III RCTs. Exclusion criteria included phase I RCTs, non-randomized controlled trials, case reports, retrospective studies, reviews, case–control studies, cross-sectional studies, abstracts, books, editorials, studies in which both the control and intervention arms contained a PARPi, and others that did not meet the inclusion criteria. 

Two authors (GCB and PS) independently screened the titles and abstracts for eligibility based on the above selection criteria. Agreed studies were then reviewed in full text independently with the above inclusion and exclusion criteria. No disagreements arose, eliminating the need for the senior author to resolve any disputes.

### 2.3. Data Extraction and Quality Assessment 

Data extraction was performed by one author (GCB) and reviewed by another (PS) for accuracy. The following were extracted from the selected studies: first author name, study name, country, publication year, Clinical Trial Identifier number (NCT), RCT trial phase, treatment arm, control arm, number of patients enrolled in each arm, median age of patients (years), median Gleason score ≥8, treatment duration (months), number of patients with homologous recombination repair (HRR) germline or somatic mutation status, number of patients with BRCA mutations, number of patients with prior taxane therapy, number of most reported side effects, number of patients who discontinued treatment or had dose reductions due to AEs, and numbers of all-grade and high-grade anemia, thrombocytopenia, and neutropenia AEs. If available, hematological toxicities were recorded using the National Cancer Institute’s Common Terminology Criteria for Adverse Events (CTCAE) [38]. Per the CTCAE 1–5 scale, “all-grade” encompasses grades 1–5, while “high-grade” includes grades 3–5. The grades correspond to the severity of the AE in chronological order. Of note, grade 5 is defined as a death related to the AE, which in this case, may not be reported. 

The risk of bias and quality assessments of the included studies were independently conducted by two authors (GCB and CH) using the revised Risk of Bias (ROB) Tool, version 2 [39]. Each study’s risk of bias was graded as low, some concerns, or high. A consensus was achieved for each study. The senior author did not need to resolve any disputes. 

### 2.4. Data Analysis

This study’s analyses were performed in Cochrane’s RevMan Review Manager 5.4.1 software (The Cochrane Collaboration, 2020) using the non-Cochrane mode. The Mantel–Haenszel method was used for dichotomous outcomes to yield risk ratios (RR) and 95% confidence intervals (CIs). Heterogeneity was quantified using *I*^2^ and considered significant when *I*^2^ > 50%. A fixed effect model was used for statistical analyses if no statistical heterogeneity existed; otherwise, a random effects model was used. 

Subgroup analyses were conducted to compare RRs of all-grade and high-grade (CTCAE ≥ 3) hematological toxicities across various factors, including the choice of PARPi, trial treatment duration (≤12 months or >12 months), PARPi monotherapy vs. combinational therapy, combination drug, and RCT trial. A *p* value of < 0.05 was considered significant. If there was a sufficient number of included studies (i.e., >10), publication bias assessment was used with Begg’s adjusted rank correlation, Egger’s linear regression, and visual inspection of the funnel plot asymmetry [40,41]. 

## 3. Results

### 3.1. Study Selection and Population Characteristics

**Figure 1:**
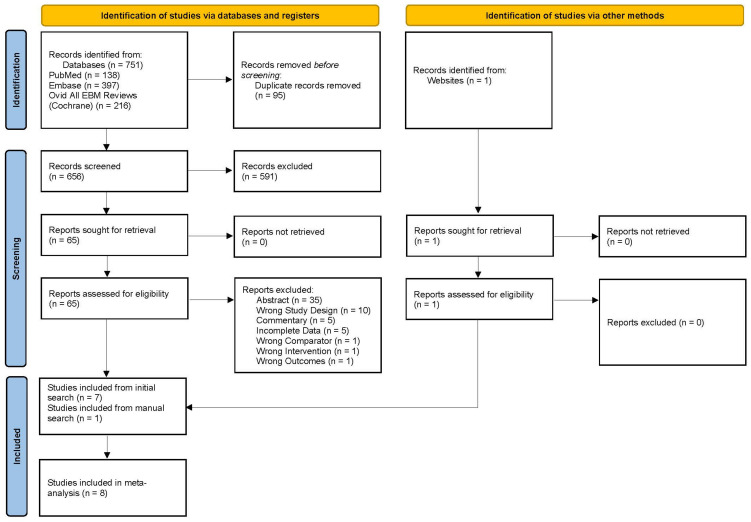
PRISMA diagram showing the study selection process, adapted from Page et al. [37].

The initial literature database query yielded a total of 751 studies from PubMed, Embase, and Ovid All EBM reviews—Cochrane, as shown in the PRISMA flow diagram in Figure 1 (adapted from Page et al. [37]). Ninety-five duplicates were removed, and after screening 656 titles and abstracts against the inclusion/exclusion criteria, 65 studies were selected for full-text review. An additional published paper was pulled from a manual search online that was not indexed in the three databases searched. In total, eight published phase II and phase III RCTs were included for the systematic review and meta-analysis [24,27,33,34,35,36,42,43]. The reasons for full-text exclusion are shown in Figure 1. Specifically, the phase II trials, such as TOPARP-A, TOPARP-B, TRITON2, TALAPRO-1, GALAHAD, QUEST, NCT02484404, CheckMate 9KD, JAVELIN PARP Medley, and TRAP, were excluded during title/abstract or full-text review due to single arm design, non-randomization protocol, or being reported in conference abstracts [17,22,44,45,46,47,48,49,50,51].

Table 1 demonstrates the main characteristics of the eight studies included in this study. PROpel II and NCI 1902 are phase II RCTs, whereas the KEYLYNK-010, MAGNITUDE, PROfound, PROpel III, TALAPRO-2, and TRITON3 are phase III RCTs [24,27,33,34,35,36,42,43]. These studies were published between 2017 and 2023 and were carried out in several institutions and countries. The studies included a variety of PARPis: Niraparib (one), Olaparib (four), Rucaparib (one), Tazaloparib (one), and Veliparib (one). The primary endpoints across the studies included imaging-based progression-free survival [24,33,34,35,36,42,43]. PSA response rate and whether ETS predicted response (imaging-based progression-free survival was a secondary endpoint) were endpoints in NCI 9012 [27]. A total of 3904 patients were recruited into these studies, of which 2218 received a PARPi and 1686 received a placebo and/or other cancer treatment. All patients had mCRPC, of which 1080 (48.7%) in the PARPi arm and 698 (41.4%) in the control arm received prior taxane therapy before enrolling in the PARPi trials. In addition, seven of the included studies reported the HRR status of patients, of which 1083 (48.8%) in the PARPi arm and 745 (44.2%) in the control arm had a genetic or tumor genomic mutation (NCI 9012 was not included in this calculation). Among those who received a PARPi, 323 (15.1%) discontinued and 522 (32.4%) reduced their PARPi dose due to an AE (NCI 9012 not included for both; KEYLYNK-010 not included for dose reduction). 

### 3.2. Risk of Bias and Quality of Evidence

The risk of bias and quality assessments are shown in Supplementary Table S3 using the revised risk of bias tool (ROB) [39]. Four studies [24,27,34,36] were open-label and considered to have high risk of bias for domain 2, whereas the other four [33,35,42,43] were double-blinded and considered to have low risk of bias. All eight studies were included in this study since the hematological toxicity outcomes are objective lab values likely to be uninfluenced by the bias above. Overall, all studies were considered to have high-quality evidence.

### 3.3. PARPi Risk of All-Grade and High-Grade Anemia

**Figure 2:**
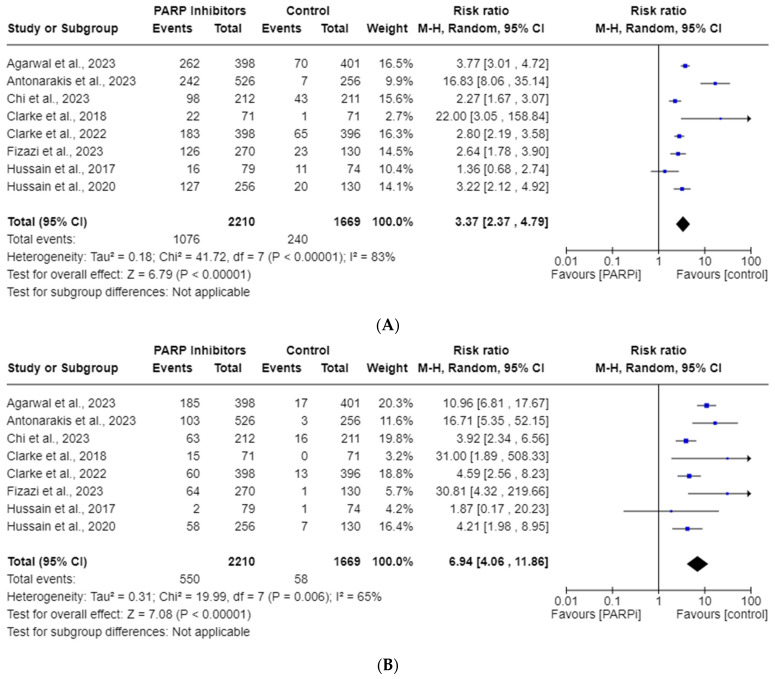
Forest plots showing the risk of all-grade (**A**) and high-grade (**B**) anemia in the PARPi RCTs [24,27,33,34,35,36,42,43].

All eight RCT studies reported anemia AEs. The total incidence of all-grade and high-grade anemia in the PARPi cohorts was 48.7% and 24.9%., respectively. Anemia was the most reported AE in five of the studies (see Table 1). Figure 2 illustrates that PARPis were significantly associated with increased risk of all-grade anemia (RR, 3.37; 95% CI, 2.37–4.79; *p* < 0.00001) and high-grade anemia (RR, 6.94; 95% CI, 4.06–11.86; *p* < 0.00001). Significant heterogeneity was observed for all-grade (*I*^2^ = 83%) and high-grade (*I*^2^ = 65%), so a random-effects model was utilized for both analyses.

### 3.4. PARPi Risk of All-Grade and High-Grade Thrombocytopenia

**Figure 3:**
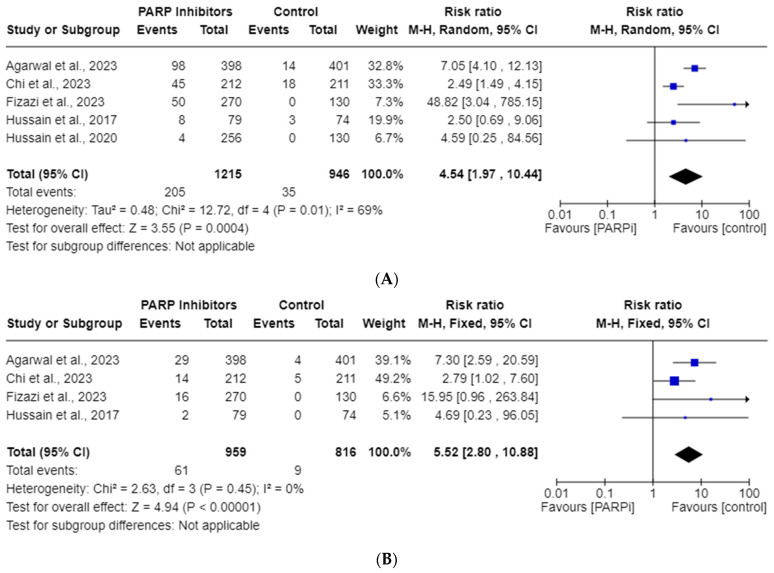
Forest plots showing the risk of all-grade (**A**) and high-grade (**B**) thrombocytopenia in the PARPi RCTs [24,27,33,35,36].

Five RCT studies were included in the all-grade, whereas only four were included in the high-grade, thrombocytopenia AE analysis. The total incidences of all-grade and high-grade thrombocytopenia in the PARPi cohorts were 16.9% and 3.70%, respectively. As shown in Figure 3, these risks of all-grade thrombocytopenia (RR, 4.54; 95% CI, 1.97–10.44; *p* = 0.0004) and high-grade thrombocytopenia (RR, 5.51; 95% CI, 2.80–10.88; *p* < 0.00001) were seen with PARPi use. Significant heterogeneity was observed for the all-grade analysis (*I*^2^ = 69%), so a random model was employed; however, it was not observed for the high-grade analysis (*I*^2^ = 0%).

### 3.5. PARPi Risk of All-Grade and High-Grade Neutropenia

**Figure 4:**
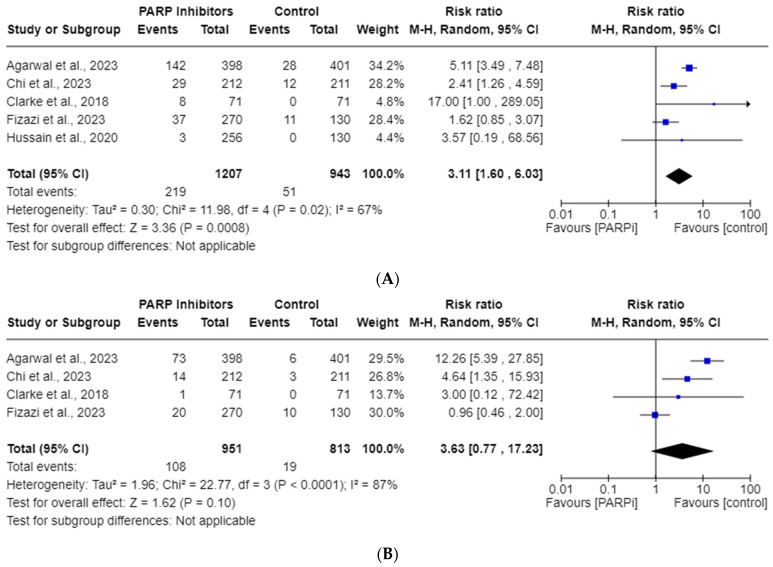
Forest plots showing the risk of all-grade (**A**) and high-grade (**B**) neutropenia in the PARPi RCTs [24,33,35,36,42].

Five studies were included in the all-grade, and four in the high-grade, neutropenia AE analyses. The total incidences of all-grade and high-grade neutropenia were 18.14% and 11.36%, respectively. Figure 4 shows this drug’s risk of all-grade neutropenia (RR, 3.11; 95% CI, 1.60–6.03; *p* = 0.0008), but highlights no high-grade neutropenia (RR, 3.63; 95% CI, 0.77–17.23; *p* = 0.10) risk. Random effects were employed due to the significant heterogeneity observed for both all-grade (*I*^2^ = 67%) and high-grade (*I*^2^ = 87%) neutropenia.

### 3.6. Subgroup Analysis

Our subgroup stratification further evaluated the hematological AEs associated with PARPis by the choice of PARPi, treatment duration (≤12 months or >12 months), PARPi monotherapy vs. combinational therapy, combination drug, and RCT trial. The RRs and *p*-values for each subgroup entity as well as the *p*-values for the overall subgroup differences are reported in Table 2 for each all-grade and high-grade hematological toxicity. In the all-grade hematological section, statistically significant differences were observed for the choice of PARPi in terms of anemia (*p* = 0.005), thrombocytopenia (*p* = 0.03), and neutropenia (*p* = 0.01). Specifically, Niraparib appeared to demonstrate the statistically lowest RRs for anemia (2.27), thrombocytopenia (2.49), and neutropenia (0.008). Further, the specific type of combination drug used in the RCTs was also significantly different for all-grade anemia (*p* < 0.0001) and thrombocytopenia (*p* = 0.005). The lowest risk for anemia (RR 2.44) and thrombocytopenia (RR 2.49) was seen in combination with Abiraterone. Otherwise, no significant subgroup differences were observed for treatment duration, monotherapy vs. combination therapy, and RCT trial. 

In the high-grade toxicities section, the PARPi of choice was significantly different for anemia (*p* = 0.004) and neutropenia (*p* = < 0.00001). Niraparib appeared to show the lowest RR statistically for high-grade anemia (5.15) and neutropenia (4.64). Significant differences were also noted in the subgroup treatment duration (*p* = 0.0004) and monotherapy vs. combination therapy (*p* = < 0.00001) for risk of neutropenia, whereas the specific type of combination drug was significant for risk of anemia (*p* = 0.002). Otherwise, the other subgroups were not found to have significant differences. 

### 3.7. Publication Bias

Given the insufficient number of included studies (i.e., <10), small effect publication bias assessment was not performed. 

## 4. Discussion

This study reports a systematic review and meta-analysis, as of 9 June 2023, on the association of all-grade and high-grade hematological toxicity side effects in mCRPC from PARPi use. While PARPis have shown favorable clinical impacts on PCa survival, they do come with adverse effects on bone marrow and often necessitate subsequent blood replacement therapies. Notably, there are no direct head-to-head efficacy trial comparisons of each PARPi in PCa. Therefore, when making shared decisions about treatment, it becomes crucial to consider the AE profile of each drug to optimize patient selection. Further, since PARPis are reserved for those with advanced disease, physicians must emphasize a balance between therapeutic benefit and potential effect of AEs on the patient’s quality of life (QoL). The primary strength of this study lies in its dedicated evaluation of hematological AEs in mCRPC, which may inform the above PARPi considerations.

Our goal was to report the incidences and RRs for anemia, thrombocytopenia, and neutropenia AEs in mCRPC patients taking PARPis. Our endpoints were recorded using the National Cancer Institute’s standardized CTCAE grading scale for AEs, with all-grade reflecting grades 1–5 and high-grade reflecting grades 3–5 [38]. Irrespective of sub-variables, the RRs for all-grade and high-grade anemia were found to be 3.37 and 6.94, respectively. For all-grade and high-grade thrombocytopenia, the RRs were 4.54 and 5.52, while for neutropenia, they were 3.11 and 3.63, respectively. All of these RRs reached statistical significance, except for high-grade neutropenia. We suspect the lack of statistical significance in high-grade neutropenia may be attributed to the limited statistical power resulting from the inclusion of only four studies in that particular analysis. Future studies may elucidate a significance in this endpoint with more data points. We suspect this would cause a regression toward the mean, but it is unclear if more pooled RCTs would significantly change the magnitude of the various RR outliers noted in Figure 2A,B, Figure 3A,B and Figure 4A. 

In our PARPi drug subgroup analyses, all of the PARPis exhibited an increase in various hematological side effects. Statistically significant subgroup differences were observed in all entries, except for high-grade thrombocytopenia. Notably, Niraparib demonstrated the least statistically significant risk for all-grade and high-grade anemia (RRs 2.27, 5.15), thrombocytopenia (RRs 2.49, 2.79), and neutropenia (RRs 2.41, 4.64). An important consideration here is that Veliparib’s RRs were the lowest in the all-grade anemia and high-grade anemia; however, these RRs did not reach statistical significance. While a head-to-head comparison does not exist, this may suggest a slightly improved hematological AE profile with Niraparib. However, these results must be interpreted with caution given the relative sample size differences in each included PARPi study. We also suspect the imbalance of Olaparib (four) vs. non-Olaparib (four) trials is likely due to the recent development and exploration of the non-Olaparib drugs compared to the well-studied Olaparib drug that has been evaluated across many cancers. Future analyses with more pooled RCTs will improve this study’s significance and better inform clinical applications. 

The subgroup analysis based on treatment duration yielded largely unremarkable results. In a prior study, Shu et al. showed a time dependency from treatment initiation, favoring an earlier onset in development of these hematological AEs [32]. Our study did not investigate this treatment initiation–time relationship, but we did not see an association with aggregate trial treatment duration. We suspect that an individual analysis (by trial participant) of these RCTs and their individual treatment timeframes may offer a conclusion consistent with the above literature. 

In the monotherapy vs. combination therapy and the specific drug combination subgroup analyses, we saw mixed results. As seen in Table 2, PARPis when used in combination showed a better profile in all-grade thrombocytopenia (RRs 2.50, 4.48) and high-grade anemia (RRs 7.15, 6.38) and thrombocytopenia (RRs 4.69, 4.79), but a worse profile in all-grade anemia (RRs 4.78, 3.07) and neutropenia (RR 4.01) and high-grade neutropenia (RR 8.43). PARPi monotherapy compared to combination therapy was noted to increase the risk of both all-grade and high-grade thrombocytopenia (RRs 26.9, 15.95). However, this same conclusion took an opposite direction for neutropenia and was unclear when applied to anemia. When looking at the specific second chemotherapy drugs used with PARPis, Abiraterone showed the lowest RRs across all all-grade and high-grade hematological toxicities. This conclusion is to be taken with caution, since RRs from Pembrolizumab combination treatment were not estimable for all-grade and high-grade thrombocytopenia and neutropenia. Lastly, the subgroup comparisons from phase II and III studies showed mixed results as well.

During our systematic review, we discovered several previously published reviews that analyzed hematological AEs in various cancers [28,30,31,52,53,54,55,56,57]. However, to the best of our knowledge, there has not been a dedicated meta-analysis investigating the hematological toxicities in PCa trials. Our study follows the consistent, reported patten of increased risk of anemia, thrombocytopenia, and neutropenia. Rizzo et al. conducted a meta-analysis of six PARPi RCT trials involving monotherapy to report an increased risk of grade 3–4 anemia AEs [54]. Only one of their included studies met the criteria for our study [23]; the other five were single-arm phase II trials [17,22,44,45,46]. Ruiz-Schutz et al. included one PCa RCT in their meta-analysis that showed increased risk of developing anemia [28,42]. Wang et al. reported an increase risk of all-grade and high-grade hematological toxicities in a 29-RCT-study review; however, their review only included two PCa studies that were combined with other non-ovarian cancers in their subgroup analysis [23,31,42]. They also suggested that combination therapy may be protective against hematological toxicities, which has been corroborated in another ovarian study [58]. However, a recent three-study meta-analysis showed non-favorable anemia AEs with PARPi combination therapy [59]. While efficacious regarding patient survival, the impact of PARPi combination therapy on hematological AEs remains controversial. 

Our meta-analysis is strengthened by several key points. First, our study’s results paint a consistent picture presented in prior hematological PARPi AEs studies [28,30,31,52,53,54,55,56,59]. Second, our study’s inclusion criteria required phase II and III RCTs to limit biases associated with non-RCTs. Third, to the best of our knowledge, this is the first dedicated study to investigate these AEs from PARPi in PCa patients. Further, our study included four studies published in the year 2023, which highlights this study’s increased data points and up-to-date nature [33,34,35,36].

This systematic review and meta-analysis is not without limitations. In the setting of strict inclusion criteria and only eight included studies, there is potential heterogeneity within this study. As with all systematic reviews, there is the possibility of “missing” a study during the literature database searches. We searched three well-known databases and conducted manual internet searches and reviewed relevant reference lists. However, we did not review the gray literature, which is another prospective source. Next, there are design and methodological differences that limit direct comparisons of each drug. With different dose administrations, reductions, treatment durations, interventions, and control groups, confounding bias cannot be ruled out. Furthermore, a few toxicity analyses, such as those of high-grade neutropenia, were limited by the availability of data. Our results suggested no increased risk of high-grade neutropenia, which is contrary to existing data [30,31]. We predict that this result is due to the lack of statistical power and would likely show regression-to-mean or an increased risk with more data points. Next, our strict inclusion/exclusion criteria aimed at reducing bias and aggregate data analyses may have generalizability concerns. Along the same lines, publication bias was also not assessed due to the low number of included studies. Additionally, we note there were more Olaparib studies compared to the other single Talazoparib, Niraparib, Rucaparib, and Veliparib studies; as such, inter-PARPi comparison results must be interpreted with caution. The onset of each toxicity and positive/negative responses to dose reduction or cessation could not be assessed in this study, unfortunately, but would be useful data for clinicians when choosing a PARPi regimen. Additionally, half of the studies were open-label trials, which introduces room for bias. As mentioned above, this bias would likely not affect their hematological profiles but may lead to patient dropout or underestimate the intention-to-treat analysis. Also, we were unable to extract data per HRR status; as such, we were unable to compare the AE incidences between HRR-positive and HRR-negative patients, which would be valuable information since the therapeutic benefit of PARPis in HRR-negative mCRPC patients is unclear. Lastly, half of the data from the phase III MAGNITUDE study were not included since they were not available at the time of the literature search [35]. Overall, the largest limitation of this study is the low number of included studies. Thus, future studies are needed when more data become available.

### 4.1. PARP Trapping—A Plausible Mechanism of Hematological Toxicities?

Although most of the PARPi trials included in this study reported similar hematological AEs, laboratory studies have suggested a differential effect in their molecular impact. A phenomenon known as “PARP trapping” may be involved in these AEs [60]. In the setting of DNA damage, PARP binds to the damaged DNA sites and initiates the synthesis of ADP-ribose polymers (PAR) that are responsible for recruiting DNA repair proteins. PARP dissociates from the DNA and allows the repair proteins access to the DNA. However, a PARPi causes the PARP to remain tightly bound to the damaged DNA (PARP trapping), not allowing the repair proteins to fix the SSBs. Consequently, SSBs can become DSBs with HRR aberrations (BRCA). It has been suggested that the PARP-2 protein might be involved in erythropoiesis, so its inhibition may be responsible for the anemia AE [61,62]. 

Murai et al. demonstrated different levels of PARP trapping potency dependent on PARPi selection—from least to most potent: Veliparib, Rucaparib, Olaparib, Niraparib, and Talazoparib [63]. Furthermore, Hopkins et al. showed that PARP trapping potency correlates with different in vivo cell toxicity levels, both in cancer and healthy bone cells [64]. Thus, this positive relationship may also explain the inverse relationship between PARP trapping potency and clinical tolerability. In our study, we did not quite see a comparable trend. From least to most, the PARPis with risk of all-grade anemia were Veliparib, Niraparib, Rucaparib, Talazoparib, and Olaparib, whereas those with the risk of all-grade thrombocytopenia were Niraparib, Veliparib, Olaparib, Talazoparib, and Rucaparib. Interestingly, Veliparib showed the least amount of risk, as well as level of PARP trapping. Further studies are needed to elucidate this relationship and determine whether PARP trapping potency plays a role in clinical toxicity profiles. 

### 4.2. Management of Hematological AEs

The included RCTs reported their individual protocols to address the hematological AEs. In general, supportive treatments (blood transfusions) were indicated if the AEs were symptomatic and/or first incidence. However, repeat episodes may have required a dose reduction or temporary cessation of the drug, as shown in Table 1. 

Chemotherapy-induced myelosuppression is a well-studied area. Ferrous sulfate, folic acid, erythropoiesis-stimulating agents, thrombopoietin, and colony stimulating factors are among many common hematological stimulating agents that have been studied to possibly address these AEs in various cancers [65,66,67,68]. Although these have yet to be explored in the PARPi-PCa setting, there is a case report of a woman using oral ferric citrate to treat her Niraparib-related anemia [69]. Also, there is another anemia case report of a patient taking Olaparib for fallopian tube cancer who was successfully treated with parental folic acid and packed red blood cell transfusions [70]. 

Each PARPi has its individual metabolic pathway. Co-administration with other drugs that alter that specific metabolic enzyme pathway may expose patients to the metabolic substrates and result in increased toxicities. For example, Olaparib is metabolized by CYP3A, and co-administration with itraconazole was noted to increase its bioavailability and serum concentration by 42%, whereas rifampin, a CYP3A4 inducer, reduced Olaparib’s concentration by 71% [71]. Therefore, there appears to be a possible advantage to reviewing a patient’s list of active drugs and cross-referencing to those with metabolic pathways similar to that of their PARPi. 

There is paucity of data on QoL in PCa patients taking PARPi. A QoL study in ovarian cancer patients showed no worsening of QoL from PARPis [72]. However, this relationship in PCa and potential positive effect of hematological prophylactic management is not quite elucidated. 

## 5. Conclusions

This systematic review and meta-analysis of eight mCRPC RCTs demonstrated an increased risk of developing anemia, thrombocytopenia, and neutropenia when using PARPis. Therefore, careful consideration of each patient’s clinical status and comorbidities must be given to avoid potential drug contraindications and subsequent high-grade AEs. We are not aware of any head-to-head comparison trials of PARPis in PCa, so physicians must pay special attention to the RCT AE profiles when selecting their patient’s therapy. Given the low number of included trials in this study, future updated pooled analyses with past, ongoing, and phase IV data will better highlight this risk of AEs in PARPis. 

## Figures and Tables

**Table 1:** Summarized Characteristics of Included RCT Studies Evaluating PARPi.

First Author	Study Name, Country	Pub. Year	NCT Number	RCT	Interventions		Sample Size		Median Age (Years)		Baseline Median Serum PSA (ug/L)		Gleason Score ≥ 8		Median Treatment Duration (Months)		HRR Mutation Status		BRCA 1/2 Mutation Status		Prior Taxane		Most Reported SE *		Drug Discontinuation *^b^		Dose Reduction *^b^	
					Interventions	Control	PARPi (n)	Control (n)	PARPi (n)	Control (n)	PARPi (n)	Control (n)	PARPi (n, %)	Control (n; %)	PARPi (n)	Control (n)	PARPi (n, %)	Control (n; %)	PARPi (n, %)	Control (n; %)	PARPi (n, %)	Control (n; %)	PARPi (n, %)	Control (n; %)	PARPi (n, %)	Control (n; %)	PARPi (n, %)	Control (n; %)
Agarwal et al. [33]	TALAPRO-2, USA ^a^	2023	NCT03395197	III	Talazoparib and Enzalutamide	Placebo and Enzalutamide	402	403	71	71	18.2	16.2	281 (70)	283 (70)	19.8	16.1	85 (21)	84 (21)	27 (7)	32 (8)	86 (21)	93 (23)	Anemia 262 (66)	Fatigue 118 (29)	76 (19)	49 (12)	223 (56)	29 (7)
Antonarakis et al. [34]	KEYLYNK-010, USA ^a^	2023	NCT03834519	III	Olaparib and Pembrolizumab	Abireterone or Enzalutamide	529	264	71	69	52.9	42.6	367 (69.4)	184 (69.7)	5	4.1	138 (26.1)	59 (22.3)	52 (9.8)	24 (9.1)	529 (100)	264 (100)	Anemia 242 (46)	Fatigue 42 (16.4)	57 (10.8)	4 (1.6)	NR	NR
Chi et al. [35]	MAGNITUDE, Canada ^a^	2023	NCT03748641	III	Niraparib and Abiraterone plus Prednisone	Placebo and Abiraterone plus Prednisone	212	211	69	69	21.4	17.4	144 (68.2)	142 (67.6)	13.8	12.1	212 (100)	211 (100)	98 (46.3)	92 (43.6)	41 (19.3)	44 (20.9)	Anemia 98 (46.2)	Hypertension/ Back Pain 44 (20.9)	23 (10.8)	10 (4.7)	42 (19.8)	7 (3.3)
Clarke et al. [42]	PROpel, UK ^a^	2018	NCT01972217	II	Olaparib and Abiraterone plus Prednisone or Prednisolone	Placebo and Abiraterone plus Prednisone or Prednisolone	71	71	70	67	86	47	NR	NR	10.3	8.4	11 (15)	10 (14)	2 (2.8)	4 (5.6)	71 (100)	71 (100)	Nausea 27 (38)	Nausea 14 (20)	21 (30)	7 (10)	13 (18)	0 (0)
Clarke et al. [43]	PROpel, UK ^a^	2022	NCT03732820	III	Olaparib and Abiraterone plus Prednisone or Prednisolone	Placebo and Abiraterone plus Prednisone or Prednisolone	399	397	69	70	17.9	16.8	265 (66.4)	258 (65)	17.5	15.7	111 (27.8)	115 (29.0)	47 (11.8)	38 (9.6)	97 (24.3)	98 (24.7)	Anemia 183 (46.0)	Fatigue 112 (28.3)	55 (13.8)	31 (7.8)	80 (20.1)	22 (5.6)
Fizazi et al. [36]	TRITON3, France ^a^	2023	NCT02975934	III	Rucaparib	Docetaxel or Abiraterone or Enzalatuamide	270	135	70	71	26.9	28.8	173 (64)	96 (71)	8.3	5.1	270 (100)	135 (100)	201 (74)	101 (75)	63 (23)	28 (21)	Fatigue 165 (61)	Fatigue 82 (63)	40 (15)	28 (22)	104 (39)	32 (25)
Hussain et al. [27]	NCI 9012, USA	2017	NCT01576172	II	Veliparib and Abiraterone plus Prednisone	Abiraterone plus Prednisone	79	74	68	69	36.4	32.7	NR	NR	9	9	UNK	UNK	UNK	UNK	23 (30.3)	16 (20.8)	Nausea 42 (53)	Fatigue 20 (27)	NR	NR	NR	NR
Hussain et al. [24]	PROfound, USA ^a^	2020	NCT02987543	III	Olaparib	Enzalutamide or Abiraterone plus Prednisone	256	131	69	69	68.2	106.5	183 (71)	95 (73)	7.6	3.9	256 (100)	131 (100)	89 (35)	52 (40)	170 (66)	84 (64)	Anemia 127 (50)	Fatigue 43 (33)	51 (20)	11 (8)	60 (23)	7 (5)

NCT, National Clinical Trials; PSA, prostate specific antigen; AE, adverse event; PARPi, Poly ADP-ribose polymerase inhibitor; NR, not reported; UNK, unknown; SE, side effect. * Numbers are based on all-grade adverse events (AEs). ^a^ Multinational trial cohorts; first author affiliation is listed. ^b^ Numbers due to AEs.

**Table 2:** Subgroup Analysis Summary for PARPi Hematological Toxicities.

All-Grade									
	Anemia			Thrombocytopenia			Neutropenia		
	RR (95% CI)	*p*-Value	*p*-Value for Overall Subgroup Differences	RR (95% CI)	*p*-Value	*p*-Value for Overall Subgroup Differences	RR (95% CI)	*p*-Value	*p*-Value for Overall Subgroup Differences
**PARPi of Choice**									
Niraparib	2.27 [1.67, 3.07]	<0.00001	0.005	2.49 [1.49, 4.15]	0.0005	0.03	2.41 [1.26, 4.59]	0.008	0.01
Olaparib	6.01 [2.51, 14.40]	<0.0001		4.59 [0.25, 84.56]	0.31		8.05 [1.04, 62.24]	0.05	
Rucaparib	2.64 [1.78, 3.90]	<0.00001		48.82 [3.04, 785.15]	0.006		1.62 [0.85, 3.07]	0.14	
Talazoparib	3.77 [3.01, 4.72]	<0.00001		7.05 [4.10, 12.13]	<0.00001		5.11 [3.49, 7.48]	<0.00001	
Veliparib	1.36 [0.68, 2.74]	0.39		2.50 [0.69, 9.06]	0.16		NE	NE	
**Treatment Duration**									
≤12 Months	4.43 [1.90, 10.32]	<0.00001	0.36	9.85 [3.33, 29.16]	<0.0001	0.18	2.73 [0.74, 10.12]	0.13	0.7
>12 Months	2.92 [2.19, 3.90]	<0.00001		4.48 [3.10, 6.46]	<0.00001		3.67 [1.76, 7.65]	0.0005	
**Monotherapy vs. Combination Therapy**									
PARPi vs. Cancer Drug	2.90 [2.17, 3.86]	<0.00001	0.91	26.90 [3.72, 194.73]	0.001	0.14	1.68 [0.90, 3.14]	0.1	0.07
PARPi plus Cancer Drug vs. Cancer Drug	4.78 [0.31, 74.80]	0.27		2.50 [0.69, 9.06]	0.16		NE	NE	
PARPi plus Cancer Drug vs. Placebo plus Cancer Drug	3.07 [2.20, 4.29]	<0.00001		4.48 [3.10, 6.46]	<0.00001		4.01 [2.00, 8.03]	<0.0001	
**Combination Drug**									
Abiraterone	2.44 [1.62, 3.68]	<0.0001	<0.0001	2.49 [1.55, 4.01]	0.0002	0.005	4.00 [0.70, 23.00]	0.12	0.79
Enzalutamide	3.77 [3.01, 4.72]	<0.00001		7.05 [4.10, 12.13]	<0.00001		5.11 [3.49, 7.48]	<0.00001	
Pembrolizumab	16.83 [8.06, 35.14]	<0.00001		NE	NE		NE	NE	
**RCT Trial**									
Phase II	3.03 [1.65, 5.58]	0.0004	0.62	2.50 [0.69, 9.06]	0.16	0.26	17.00 [1.00, 289.05]	0.05	0.28
Phase III	3.55 [3.11, 4.04]	<0.00001		5.38 [3.73, 7.76]	<0.00001		3.57 [2.69, 4.74]	<0.00001	
**High-Grade**									
	**Anemia**			**Thrombocytopenia**			**Neutropenia**		
	**RR (95% CI)**	***p*** **-Value**	***p*** **-Value for Overall Subgroup Differences**	**RR (95% CI)**	***p*** **-Value**	***p*** **-Value for Overall Subgroup Differences**	**RR (95% CI)**	***p*** **-Value**	***p*** **-Value for Overall Subgroup Differences**
**PARPi of Choice**									
Niraparib	5.15 [2.86, 9.28]	<0.00001	0.004	2.79 [1.02, 7.60]	0.05	0.48	4.64 [1.35, 15.93]	0.01	0.0001
Olaparib	8.21 [3.76, 17.91]	<0.00001		NE	NE		3.00 [0.12, 72.42]	0.5	
Rucaparib	40.08 [5.49, 292.44]	0.0003		15.95 [0.96, 263.84]	0.05		0.96 [0.46, 2.00]	0.92	
Talazoparib	19.62 [11.62, 33.14]	<0.00001		7.30 [2.59, 20.59]	0.0002		12.26 [5.39, 27.85]	<0.00001	
Veliparib	1.90 [0.17, 21.36]	0.6		4.69 [0.23, 96.05]	0.32		NE	NE	
**Treatment Duration**									
≤12 Months	10.22 [5.82, 17.93]	<0.00001	0.19	11.07 [1.37, 89.44]	0.02	0.46	1.02 [0.5, 2.08]	0.96	0.0004
>12 Months	6.70 [4.98, 9.02]	<0.00001		4.79 [2.36, 9.73]	<0.0001		8.43 [3.31, 21.49]	<0.00001	
**Monotherapy vs. Combination Therapy**									
PARPi vs. Cancer Drug	9.59 [1.12, 82.12]	0.04	0.94	15.95 [0.96, 263.84]	0.05	0.72	0.96 [0.46, 2.00]	0.92	<0.0001
PARPi plus Cancer Drug vs. Cancer Drug	7.15 [0.83, 61.45]	0.07		4.69 [0.23, 96.05]	0.32		NE	NE	
PARPi plus Cancer Drug vs. Placebo plus Cancer Drug	6.38 [3.29, 12.39]	<0.00001		4.79 [2.36, 9.73]	<0.0001		8.43 [4.11, 17.32]	<0.00001	
**Combination Drug**									
Abiraterone	4.27 [2.93, 6.23]	<0.00001	0.002	2.96 [1.15, 7.67]	0.03	0.21	4.41 [1.40, 13.89]	0.01	0.16
Enzalutamide	10.96 [6.81, 17.67]	<0.00001		7.30 [2.59, 20.59]	0.0002		12.26 [5.39, 27.85]	<0.00001	
Pembrolizumab	16.71 [5.35, 52.15]	<0.00001		NE	NE		NE	NE	
**RCT Trial**									
Phase II	11.38 [2.20, 58.69]	0.004	0.63	4.69 [0.23, 96.05]	0.32	0.91	3.00 [0.12, 72.42]	0.5	0.9
Phase III	7.52 [5.74, 9.86]	<0.00001		5.57 [2.78, 11.16]	<0.00001		3.75 [0.65, 21.68]	0.14	

NE, not estimable.

## Data Availability

Data collected and used for this study are available in the referenced trials.

## Supplementary Materials

The following supporting information can be downloaded at: https://www.mdpi.com/article/10.3390/cancers15194904/s1, Table S1: PRISMA Checklist; Table S2: Literature Search Strategies per Database; Table S3: RCT Quality Evaluation using the Cochrane Risk of Bias (ROB).
